# An Approach for Measuring the Dielectric Strength of OLED Materials

**DOI:** 10.3390/ma11060979

**Published:** 2018-06-09

**Authors:** Sujith Sudheendran Swayamprabha, Deepak Kumar Dubey, Wei-Chi Song, You-Ting Lin, Rohit Ashok Kumar Yadav, Meenu Singh, Jwo-Huei Jou

**Affiliations:** Department of Materials Science and Engineering, National Tsing Hua University, Hsinchu 30013, Taiwan; sujithsudheendran.s.s@gmail.com (S.S.S.); deepak08gkp@gmail.com (D.K.D.); ahenry94125@gmail.com (W.-C.S.); zaa789p2002@hotmail.com (Y.-T.L.); rohitakyadav@gmail.com (R.A.K.Y.); meenu9696@gmail.com (M.S.)

**Keywords:** Breakdown voltage, Dielectric strength, OLED

## Abstract

Surface roughness of electrodes plays a key role in the dielectric breakdown of thin-film organic devices. The rate of breakdown will increase when there are stochastic sharp spikes on the surface of electrodes. Additionally, surface having spiking morphology makes the determination of dielectric strength very challenging, specifically when the layer is relatively thin. We demonstrate here a new approach to investigate the dielectric strength of organic thin films for organic light-emitting diodes (OLEDs). The thin films were deposited on a substrate using physical vapor deposition (PVD) under high vacuum. The device architectures used were glass substrate/indium tin oxide (ITO)/organic material/aluminum (Al) and glass substrate/Al/organic material/Al. The dielectric strength of the OLED materials was evaluated from the measured breakdown voltage and layer thickness.

## 1. Introduction

Organic light-emitting diodes (OLEDs) have drawn enormous attention in both academia and industry owing to their cumulative applications in high-quality flat-panel displays and solid-state lighting [1,2,3,4,5,6]. Recently, OLEDs have been commercialized as information displays for car audio systems, sub-displays of cellular phones, and large-screen TVs, promising large market opportunities [7,8,9,10]. The superior properties of OLEDs start to dominate existing flat-panel display technology, liquid-crystal display (LCD). OLEDs have striking topographies, such as high color purity, high luminance, a wide viewing angle, high contrast and response speed, low power consumption, a simple fabrication process, ultra-thin structure, light weight, flexibility, and low cost [11]. To make OLED displays and lighting more competitive and customer affordable, and the resultant products more energy saving and longer lasting, OLEDs with higher power efficiency are demanded [3].

In the last two decades, state-of-the-art OLEDs have achieved prodigious progress in efficiency, making their potential applications exceedingly promising [3]. However, lifespan is still a crucial reliability issue to be addressed before they can be extensively adopted. Light-emitting diodes based on both inorganic and organic semiconducting materials suffer from internal energy losses, and these losses convert into heat [12]. The produced heat can then itself act as a source of degradation and decrease the lifetime of the device. Therefore, reduction of heat and dissipation is required in order to increase the lifetime of OLEDs [13]. In addition, device failures and lifetime are affected by several other factors, such as electrochemical degradation, oxidation, moisture, molecular migrations, and dielectric breakdown [12]. Surface roughness of the electrodes plays a major role in dielectric breakdown [14].

Only a few reports are available regarding the dielectric properties of OLED materials. In 1993, Nguyen et al. analyzed frequency-dependent capacitive response in indium tin oxide (ITO)/poly(p-phenylene vinylene) (PPV)/aluminum (Al) devices, such as conductance and tan δ [15]. Li et al. studied the alternating current impedance of light-emitting diodes (LED) and light-emitting electrochemical cells (LEC), and propsed an equivalent circuit with a series combination of series resistance (R_0_) and parallel resistor-capacitor (RC) [16]. Even though the structure of OLEDs has a single layer, it shows two semicircles in Cole–Cole plot. Wang et al. reported that materials with high dielectric constant can perform as a good electron injection layer (EIL), which enhanced device efficiency and brightness [17]. Ohta et al. studied the influence of high dielectric strength materials in an active matrix driving OLEDs [18]. Ahn et al. examined the magnitude and phase of impedance, electrical conductivity, and dielectric loss under different biasing voltage in OLEDs [19].

Generally, OLED devices have a driving voltage of 3 to 5 V. Depending on the color of the light and the thickness of organic layers, OLED devices need higher voltage in order to achieve more brightness. However, device efficiency and lifetime start to decline at a higher driving voltage. This failure of OLED devices is mainly due to dielectric breakdown of the organic material. For a thin film of 100 nm, a voltage of 10 V can produce an electric field of 1 million volts/cm (MV/cm). The produced electric field is sufficient to cause dielectric breakdown of most of the OLED materials [20]. In this study, we attempted to discover the correlation between surface roughness of the electrode and dielectric breakdown of OLED materials. We measured the dielectric strength of the OLED materials by evaluating the breakdown voltage and layer thickness [21].

## 2. Experimental Section

### 2.1. Materials

**Figure 1:**
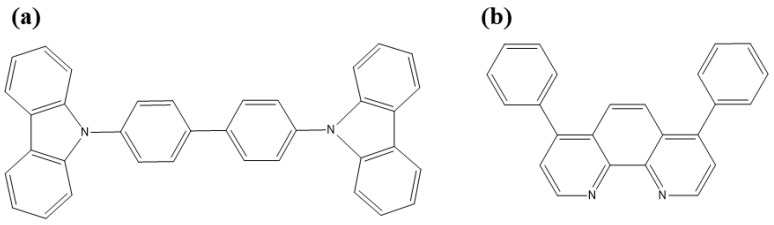
Molecular structures of (**a**) 4,4′-Bis(9-carbazolyl)-1,1′-biphenyl,4,4-*N*,*N*′-Dicarba zole-1,1′biphenyl (CBP) and (**b**) 4,7-Diphenyl-1,10-phenanthroline (BPhen).

Indium tin oxide (ITO) coated glass substrates with a sheet resistance of 15 Ω/sq, surface roughness of 1.5 nm, and light transmittance greater than 84% were purchased from Luminescence Technology Corporation, Hsinchu, Taiwan. The molecular structures of the organic materials used in this work are shown in Figure 1. The sublimated grade organic materials 4,7-Diphenyl-1,10-phenanthroline (BPhen) and 4,4′-Bis(9-carbazolyl)-1,1′-biphenyl,4,4-*N*,*N*′-Dicarba zole-1,1′biphenyl (CBP) were purchased from the Luminescence Technology Corporation and Wang Shine Co., Taichung, Taiwan, respectively. Aluminum (Al) ingots (99.999%) were purchased from Showa Chemical Co. Ltd., Tokyo, Japan. All materials were used without any further purification.

### 2.2. Device Fabrication

The study of the dielectric properties of OLED devices was performed on the organic layer sandwiched between two electrodes, with the device structure metal/organic layer/metal (MOM). The devices were grown on glass slides pre-coated with ITO. The ITO substrates were ultra-sonicatied sequencially in acetone and isopropanol for 30 min, followed by 20 min of ultraviolet ozone treatment to eliminate all organic impurities. Device I was fabricated with an architecture of Glass/ITO/CBP/Al; Device II: Glass/Al/CBP/Al; Device III: Glass/ITO/BPhen/Al; and Device IV: Glass/Al/BPhen/Al. All materials were subsequently deposited on the substrates by thermal evaporation under high vacuum (<10^−6^ torr). Device I comprised of a 1250 Å ITO anode, 1200 Å CBP, and a 1000 Å aluminum cathode. Device II comprised of a 1250 Å aluminum anode, 1200 Å CBP, and a 1000 Å aluminum cathode. Device III and Device IV followed the same thickness of the materials of Device I and Device II, respectively, but replacing CBP with BPhen. All thickness measurements were done by alpha step (Dektak 150, Veeco Instruments, Inc., Plainview, NY, USA) where possible instrumental error may be ±5 nm. Purity of the deposited organic materials CBP and BPhen were confirmed by proton nuclear magnetic resonance (^1^HNMR, Bruker Avance 400 NMR spectrometer, Bruker Corp., Billerica, MA, USA), as shown in Figures S1 and S2, respectively. 

## 3. Measurements

Surface morphology was studied using an atomic force microscope (Digital Instruments Nanoscope IIIa, Bruker Corp., Billerica, MA, USA) in the tapping mode. A Keithley 2400 electrometer (Keithley Instruments, Inc., Cleveland, OH, USA) was used to measure the current-voltage (I-V) characteristics. All electrical characterizations have been done under open environment at room temperature.

From the breakdown voltage, we calculated the dielectric strength of the material using the equation:

(1) E=V/t

where, *E*, *V*, and *t* are the dielectric strength, breakdown voltage in MV, and deposited layer thickness in cm, respectively.

## 4. Result and Discussion

**Figure 2:**
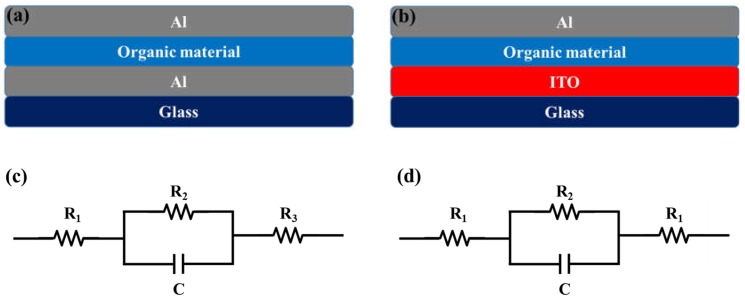
(**a**,**b**) show the studied device architectures for the dielectric strength measurements and (**c**,**d**) show their respective electrical circuit diagrams.

**Figure 3:**
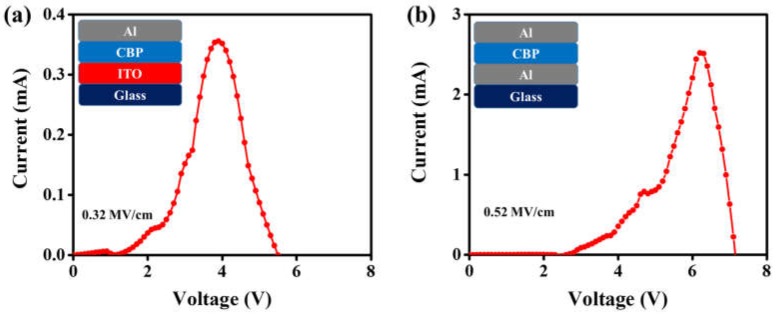
Current-Voltage (I-V) characteristics of the devices having 120 nm CBP layer with (**a**) ITO/CBP/aluminum (Al) and (**b**) Al/CBP/Al device structures.

Figure 2a,b shows the studied devices composed with glass/ITO/organic material/Al and glass/Al/organic material/Al for dielectric strength measurement, respectively. Figure 2c,d illustrates the respective electric circuits of studied devices. Figure 3a,b shows the breakdown voltage of Device I and Device II. Device I showed a breakdown voltage of 3.90 V, whereas Device II showed 6.20 V. The calculated dielectric strengths were 0.32 and 0.52 MV/cm for Device I and Device II, respectively.

**Figure 4:**
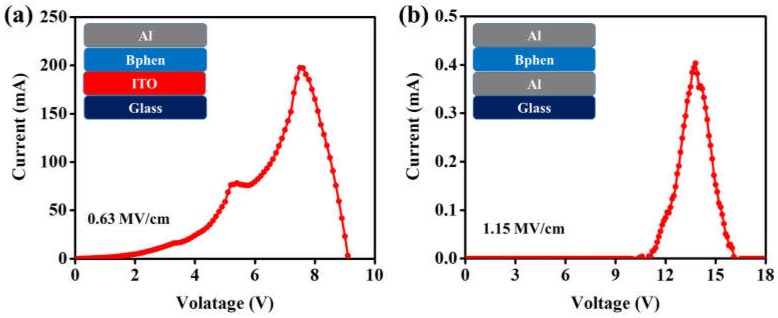
Current-voltage (I-V) characteristics of the devices having a 120 nm BPhen layer with (**a**) Al/BPhen/Al and (**b**) ITO/BPhen/Al device structures.

Similarly, Figure 4a,b shows the breakdown voltages of Device III and Device IV. Device III showed a lower breakdown voltage when compared with Device IV. The breakdown voltages were 7.52 and 13.80 V for Device III and Device IV, respectively. The calculated dielectric strengths were 0.63 and 1.15 MV/cm for Device III and Device IV, respectively. The dielectric strength results are summarized in Table 1.

**Figure 5:**
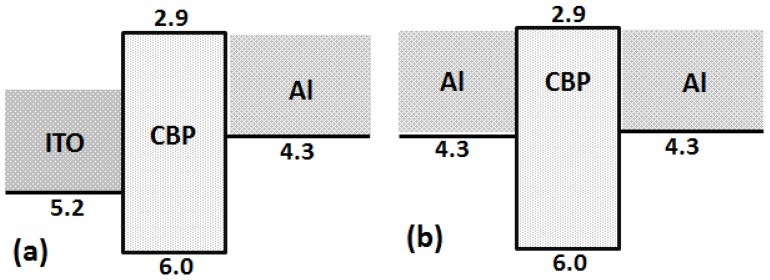
Schematic energy diagram of devices with the following device structure: (**a**) ITO/CBP/Al; and (**b**) Al/CBP/Al.

**Figure 6:**
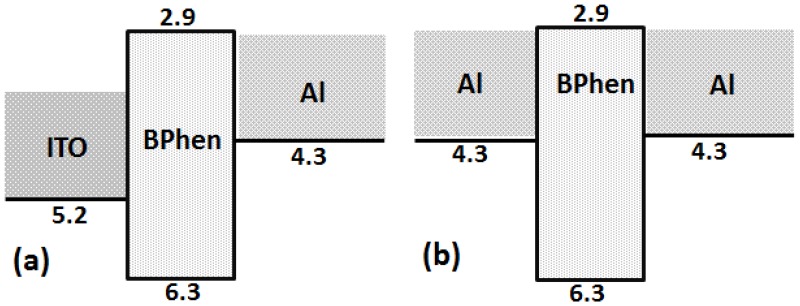
Schematic energy diagram of devices with the following device structure: (**a**) ITO/BPhen/Al; and (**b**) Al/BPhen/Al.

The reason why devices fabricated with an ITO anode show low dielectric strength when compared with devices with an Al anode may be attributed to two important factors in the designed device architecture. First, the Al anode has a low work function (4.3 eV) as compared to the ITO anode (5.2 eV). Second, the devices fabricated with an ITO anode more effectively transfer holes into the organic layer because of 0.8 and 1.1 eV hole-injection barriers at the interface of ITO/CBP and ITO/BPhen, respectively, i.e., 1.7 and 2.0 eV for the devices with Al as an anode, as shown in Figure 5a,b and Figure 6a,b . Furthermore, we observed the low charge injection region in Figure 3b and Figure 4b, below the voltage 2 V and 10 V, for Devices II and IV, respectively. That current density (J) follows the externally applied voltage (V) linearly, as shown in Equation (2), may be the reason behind this [22].

(2) $$J=en{µ}_{eff}\frac{V}{L}$$

where *en* is the charge density (*e*: elementary charge, *n*: charge carrier density), μ*_eff_* is the effective charge mobility, and *L* the organic film thickness. It is notable that the effective charge mobility μ*_eff_* includes charge trapping phenomenon as follows [22]:

(3) $${µ}_{eff}={µ}_{0}\left(\frac{n_{mob}}{n_{mob}+n_{trap}}\right)$$

where μ_0_ is trap-free charge mobility, and *n_mob_* and *n_trap_* are mobile and trapped charge carrier densities, respectively. As shown in Figure 3b, in the voltage region from 2 to 3 V an abrupt increase of the current is observed. Note that the significant rise of the current in certain voltage regions only is usually assigned to the trap-filled-limit (TFL) voltage. In other words, the charge transport properties of organic films are changed due to filling of all localized states, and charge carriers are no more influenced by the trapping mechanism. Similar behavior is also observed in Device IV in the voltage region from 10 to 12 V, as shown in Figure 4b.

In addition, the devices with BPhen as an organic layer show higher dielectric strength as compared to devices with CBP organic layers. The reasons behind this may be: (i) highest occupied molecular orbital (HOMO) level of BPhen is deeper (6.3 eV); (ii) high electron mobility of BPhen; and (iii) the bipolar nature of CBP [23,24,25,26].

**Figure 7:**
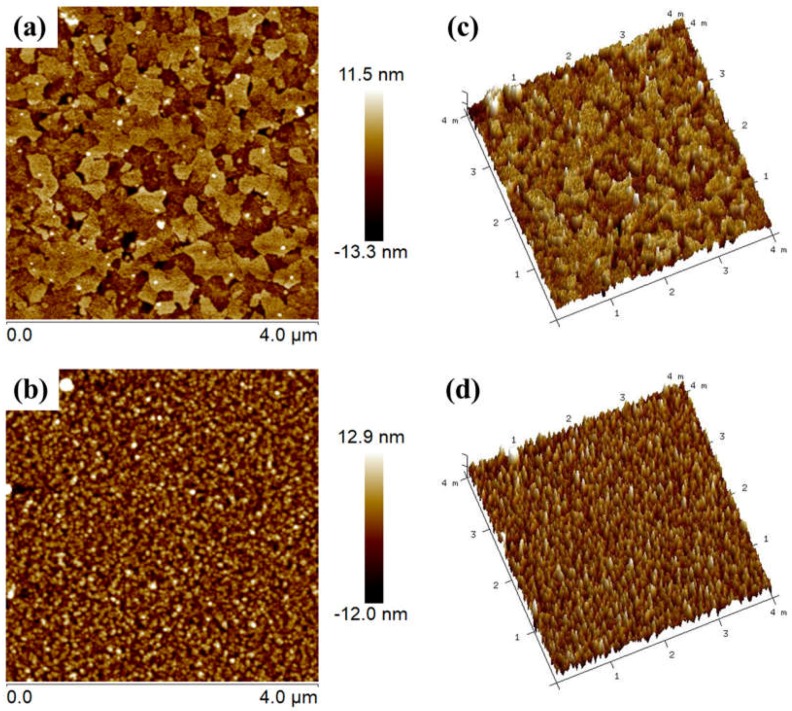
(**a**,**b**) show the 2D atomic force micrographs of the ITO and thermally deposited Al anode surface, respectively; (**c**,**d**) show their respective 3D micrograps.

The surface topography of the pristine ITO and thermally deposited Al were studied using atomic force microscopy (AFM) in the tapping mode. AFM data analysis provides quantitative information about surface morphology. Two-dimensional (2D) and three-dimensional (3D) AFM micrographs of the pristine ITO and thermally deposited Al (Figure 7) samples clearly show that the obtained films indeed possess a uniform surface, very developed grain boundaries, and free surfaces. Roughness of the films was estimated with Nanoscope analyser. Scans over 4 × 4 µm^2^ were taken in order to measure the surface roughness of the films. The root-mean square roughness (*R_RMS_*) was estimated by using the formula [27]:

(4) $$R_{RMS}=\overset{N}{\underset{i=1}{\sum }}{\left(\frac{{\left(h_{i}-ћ\right)}^{2}}{N}\right)}^{1/2}$$

where *h_i_* and *ħ* represents the height value at each data point and the profile mean value of the surface, respectively, and *N* is the number of data points in the analyzed profile. The standard roughness, or the arithmetic average roughness–height, represents the arithmetic mean of the deviations in height from the profile mean value, where the profile mean value is defined as [27]:

$$ћ=\frac{1}{N}\overset{N}{\underset{i=1}{\sum }}h_{i}$$

The *R_RMS_* estimated for the films of thermally deposited Al and pristine ITO were 2.5 and 3.6 nm, respectively. We could correlate the variation in dielectric strength with the surface roughness of both ITO and Al. The dielectric strength of materials was inversely proportional to the surface roughness of the anode. The reason why devices with an ITO anode show lower dielectric strength as compared to devices with an Al anode may also be attributed to the low surface roughness of Al as compared to ITO.

## 5. Conclusions

In this study, the dielectric properties of OLED materials were investigated using metal/organic layer/metal device structures. The surface roughness of electrodes and dielectric strength of organic materials play a crucial role in dielectric breakdown, which is directly related to the performance of OLED devices. Surface roughness of electrodes will accelerate the dielectric breakdown. By optimizing the surface roughness of electrodes and the dielectric strength of organic materials, we can design and fabricate OLEDs with long lifetime and high-efficiency. Despite various advantages in OLEDs, a fundamental research of their physical properties is not yet been fully explored. Our findings might help domain experts carry out extensive studies to design high-efficiency and long lifespan devices.

## Figures and Tables

**Table 1:** Tabulation of the used anode, observed breakdown voltage, and calculated dielectric strength for the deposited 120 nm thickness of the organic layer.

Organic Material	Anode	Breakdown Voltage (V)	Calculated Dielectric Strength (MV/cm)
CBP	ITO	3.90	0.32
CBP	Al	6.20	0.52
Bphen	ITO	7.52	0.63
Bphen	Al	13.80	1.15

## Supplementary Materials

The following are available online at http://www.mdpi.com/1996-1944/11/6/979/s1, Figure S1: ^1^H NMR analysis of 4,4′-Bis(9-carbazolyl)-1,1′-biphenyl,4,4-*N*,*N*′-Dicarbazole-1,1′biphenyl (CBP). Figure S2: ^1^H NMR analysis of 4,7-Diphenyl-1,10-phenanthroline (BPhen).
