# Supplementary material for: An Approach for Measuring the Dielectric Strength of OLED Materials
Source: Materials (Basel). 2018 Jun 9;11(6):979. doi: 10.3390/ma11060979 (PMC6025577; doi:10.3390/ma11060979)
Supplement: Supplementary file 1 [file materials-11-00979-s001.pdf]

CBP

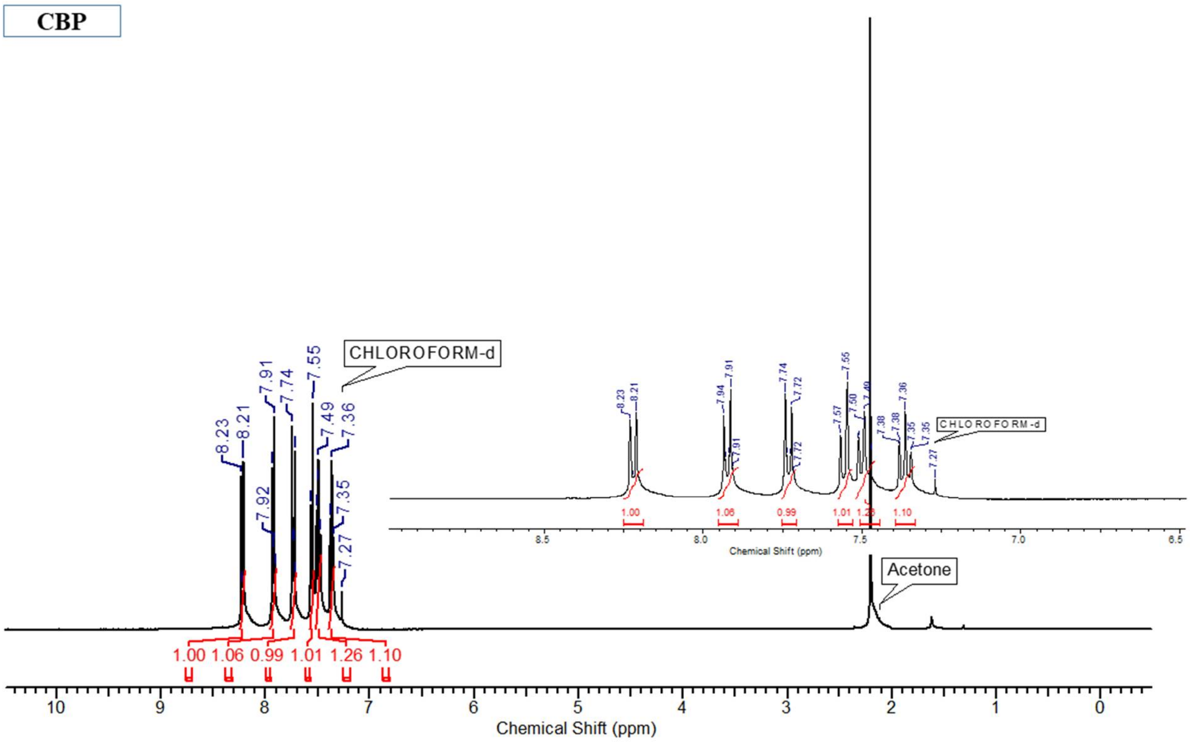

Fig. S1 ^{1}H NMR analysis of 4,4′-Bis(9-carbazolyl)-1,1′-biphenyl,4,4-N,N′-Dicarbazole-1,1′biphenyl (CBP)

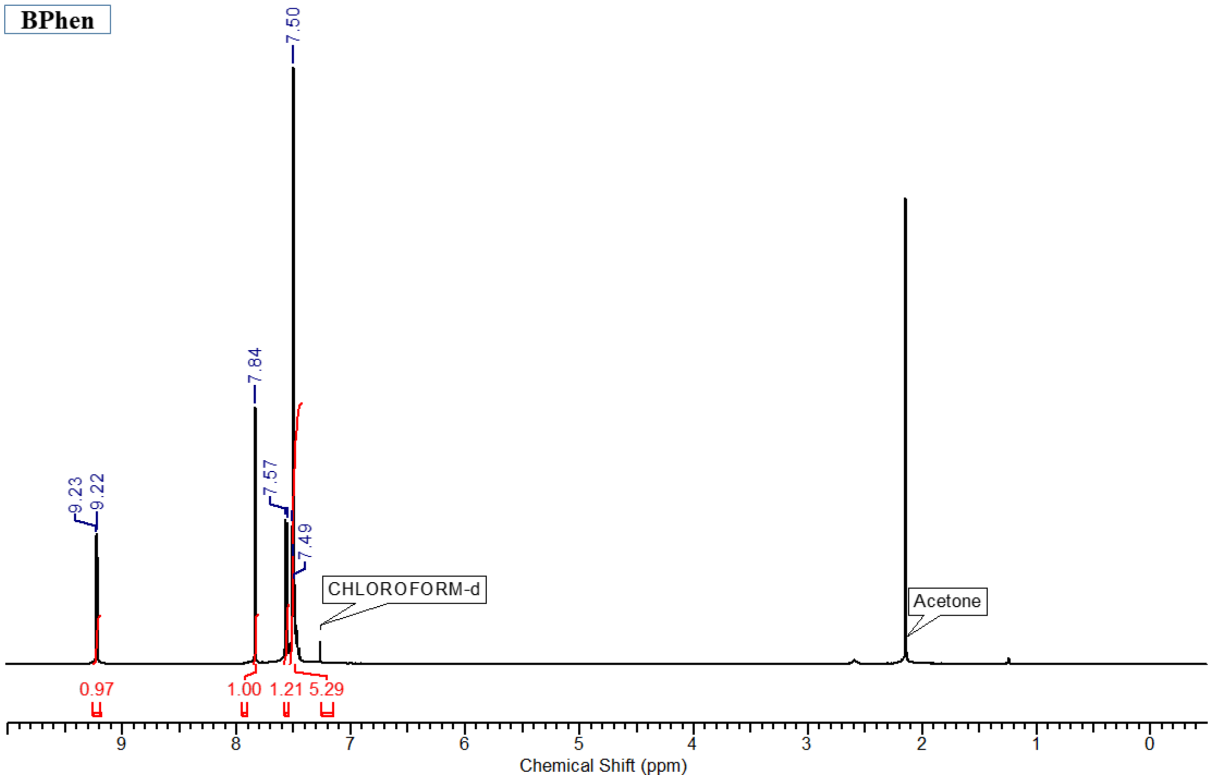

Fig. S2 ^{1}H NMR analysis of 4,7-Diphenyl-1,10-phenanthroline (BPhen)
